# Genetic diversity with potential ESBL-producing and multidrug-resistant *Salmonella* strains from chicken meat

**DOI:** 10.3389/fvets.2025.1709758

**Published:** 2026-01-13

**Authors:** Selman Bahadır Orhan, Ali Anil Suleymanoglu, Ali Aydin

**Affiliations:** Department of Food Hygiene and Technology, Faculty of Veterinary Medicine, Istanbul University-Cerrahpasa, Avcilar, Istanbul, Türkiye

**Keywords:** chicken meat, extended-spectrum beta-lactamase, multidrug resistance, *Salmonella* serotypes, sanger sequence

## Abstract

**Objectives:**

*Salmonella*, a major foodborne pathogen, is a primary concern due to its role in spreading antibiotic resistance. Raw chicken meat samples (*n* = 210) were collected from various retail locations in Istanbul.

**Methods:**

The food samples were isolated according to ISO 6579-1 and 13 (6.2%) of *Salmonella* strains confirmed through PCR, agglutination tests, and Sanger sequencing; *S.* Infantis (84.6%) was identified as the dominant type. The other types found included *S.* Enteritidis (7.7%) and *S.* Virchow (7.7%). Additionally, antibiotic susceptibility was tested according to EUCAST and CLSI standards in different *Salmonella* serotypes. The serotypes were analyzed for susceptibility to 13 antibiotics using agar-disk diffusion assays, and resistance levels were determined via E-test.

**Results:**

The disc diffusion method revealed resistance to cefazolin across all *Salmonella* serotypes. High resistance rates were also observed for pefloxacin (84.6%), azithromycin (76.9%), and tetracycline (84.6%). Multidrug resistance was identified in 11 (84.6%) strains by the disc diffusion test. The minimum inhibitory concentration testing with MIC test strips showed high tetracycline resistance at 84.6%. The *bla*_TEM_ gene was found in 30.7% of strains, while *bla*_CTX-M_ subgroup 1 (7.7%) and *bla*_CTX-M_ subgroup 9 (30%) were detected by multiplex PCR; however, and *bla*_CTX-M_, *bla*_OXA-2_, and *bla*_SHV_ genes were not present. Resistance to carbapenem and colistin was also checked via PCR, and *bla*_OXA-48_, *bla*_VIM_, *bla*_NDM_, *bla*_KPC_, and *mcr* genes were not detected in the Salmonella serotypes.

**Conclusion:**

This pioneering study provides a comprehensive analysis of serotyping and ESBL production in Salmonella strains isolated from Istanbul.

## 1 Introduction

*Salmonella* (*S*.), a major foodborne pathogen, is a leading cause of diarrheal diseases worldwide. It can be present at all stages of the food supply chain and may contaminate food products through cross-contamination. Furthermore, the development of antibiotic resistance in *Salmonella*, which can endure various conditions, represents a significant and urgent public health threat. The rise of antimicrobial resistance makes it increasingly difficult to treat salmonellosis symptoms. Food products such as poultry, meat, eggs, and milk (1, 2) are primary sources of salmonellosis in humans. However, poultry remains *Salmonella*’s main reservoir, posing a threat to food safety through eggs and chicken meat (3, 4).

Chicken meat is one of the world’s most popular and preferred animal protein sources. Poultry is also the second most produced and consumed meat in Europe, with remarkable consumption in countries such as England, France, Spain, Ireland, and Portugal (5). However, Türkiye is the world’s seventh-largest exporter, with poultry meat production expected to reach 2,245,770 tons in 2021 (6). Indeed, worldwide production and consumption of fresh poultry meat are projected to increase significantly in 2024, with a 3.5% increase compared to poultry production and consumption in 2023 (7). Nevertheless, according to the “One Health 2021 Zoonoses Report” of EFSA and ECDC (8), the prevalence of *Salmonella* was reported as 7.3% in fresh poultry meat tested and offered for retail sale and 7.6% in meat products made from poultry meat and intended for consumption, cooked. On the other hand, the distribution of *Salmonella* serotypes in chicken meat has changed compared to previous studies (3, 9).

Antibiotic resistance increasingly threatens human and animal health. Due to the uncontrolled and overuse of antibiotics, microorganisms resistant to various antibiotics are identified daily. The World Health Organisation (WHO) states that antibiotic resistance is one of the most critical threats to humanity (10). *Enterobacterales*, including *Salmonella*, are a significant public health threat due to the development of antibiotic resistance today (11). The WHO ranks pathogenic bacteria for public health according to the importance of antibiotic resistance, and fluoroquinolone-resistant *Salmonella* is in the high-importance group (12). Moreover, *Salmonella* spp., *Campylobacter* spp., indicator *Escherichia coli* (*E. coli*), and methicillin-resistant *Staphylococcus aureus* are among the antibiotic-resistant zoonotic and indicator bacteria of food, human, and animal origin that pose a threat in the European Union (EU) (13). Various antibiotic-resistant *Salmonella* serotypes have been reported by many researchers (14–16).

ESBL-producing Enterobacterales present a major global public health threat. Last-resort antibiotics like carbapenems and colistin are used to fight this, especially when there’s resistance to 3rd-generation cephalosporins. ESBLs make antibiotics such as penicillins, monobactams, and cephalosporins ineffective, which are commonly used to treat diseases in humans and animals. They can also break own carbapenem antibiotics, though the effectiveness of carbapenems is evaluated separately. Carbapenem-resistant Enterobacterales and those resistant to 3rd-generation cephalosporins are classified as critical pathogens by WHO (12). Consequently, colistin—one of the few remaining treatment options, particularly against carbapenem-resistant bacteria—becomes a vital focus (11).

The aim of this study was (a) to examine the presence of *Salmonella* in chicken meat samples collected in the Asian and European parts of Istanbul with conventional methods and PCR; (b) serotyping of *Salmonella* isolates by sanger sequence and detection of phylogenetic affinities; (c) to determine the antibiotic susceptibility against 9 antibiotics groups (aminoglycosides, cephalosporins, macrolides, fluoroquinolones, penicillin, phenicol, sulfonamides, tetracyclines and carbapenems) by agar-disc diffusion assays according to the EUCAST and CLSI standards and detection Minimum Inhibitory Concentration (MIC) value of resistant *Salmonella* serotypes using E-test, and (d) to investigate phenotypic ESBL production and the ESBLs genes (*bla*_TEM_, *bla*_CTX-M_ subgroup (1–2–8-9-25/26), *bla*_SHV_ and *bla*_OXA-2_), the carbapenem (*bla*_OXA-48_, *bla*_VIM_, *bla*_NDM_ and *bla*_KPC_) and mobilized colistin (*mcr*-1, *mcr*-2, *mcr*-3, *mcr*-4, and *mcr*-5) as resistance genes in *Salmonella* serotypes by PCR.

## 2 Materials and methods

### 2.1 Sampling

A total of 210 raw chicken meat samples were collected from different sale points in Istanbul, Türkiye, between May and August 2021. The samples from the European part of Istanbul included drumsticks (*n* = 15), breasts (*n* = 25), thighs (*n* = 26), and wings (*n* = 40). From the Asian part, the samples consisted of drumsticks (*n* = 15), breasts (*n* = 33), thighs (*n* = 27), and wings (*n* = 29). All samples were transported under cold conditions (≤ + 4 °C) in thermal boxes to the Department of Food Hygiene and Technology at Istanbul University-Cerrahpasa immediately after collection.

### 2.2 Isolation and identification of *Salmonella* by conventional methods

*Salmonella* was isolated and identified according to the ISO 6579-1 standard method (17). 25 g of the collected raw chicken meat samples and 225 mL of buffer peptone water (Oxoid CM 0509, Basingstoke, United Kingdom) were added and homogenized in a stomacher (Interscience, Saint Nom la Bretèche, France). Subsequently, 1 mL of inoculum was transferred into 10 mL Mueller Kaufmann Tetrathionate Broth (MKTTn) (Oxoid, CM1048), and 0,1 mL of inoculum from stomacher bags was transferred to 10 mL Rappaport-Vassiliadis Broth (Oxoid, CM0866). The MKTTn and RVS broth were incubated at 37 °C and 41.5 °C for 24 ± 3 h, respectively. Subsequently, the samples were inoculated on Xylose Lysine Desoxycholate (Oxoid, CM0469) and Hectoen Enteric Agar (Oxoid, CM0419) and incubated at 37 °C for 24 h. Suspected *Salmonella* isolates were inoculated on Nutrient agar (Millipore, 105,450) for purification and incubated at 37 °C for 24 h. The purified colonies were stored in Tryptone Soya Broth (TSB) (Oxoid, CM0129) with 20% glycerol at −18 °C.

For biochemical confirmation, suspected *Salmonella* colonies were transferred to Triple Sugar Iron Agar (Oxoid, CM0277), Lysine Iron Agar (Oxoid, CM0381), and Urea Broth (Oxoid, CM0071) media and incubated at 37 °C for 24 h (17). Subsequently, isolated *Salmonella* spp. were confirmed by PCR, agglutination test, and Sanger sequencing.

In the analysis, *S. typhimurium* ATCC 14028, *S.* Enteritidis ATCC 13076, and *S.* Infantis ATCC 51741 were used as positive controls, and while *E. coli* ATCC 25922 was used as a negative control.

### 2.3 Verification of *Salmonella* isolates by PCR

#### 2.3.1 DNA extraction

The DNA extraction method of was used to extract genomic DNA of suspected *Salmonella* isolates according to Liu et al. (18). Epoch2 (BioTek, United States) tested the acquired DNA for quality and stored at −20 °C.

#### 2.3.2 Confirmation of *Salmonella* isolates by PCR

The identification of *Salmonella* spp. was performed using PCR. invA F-(5′- GTGAAATTATCGCCACGTTCGGGCAA-3′) and *inv*A R-(5′- TCATCGCAC-CGTCAAAGGAACC-3′) (284 bp), which are specific primers for *invA (Salmonella-specific gene)* were used in PCR. The PCR assay was conducted with the following conditions: initial denaturation at 72 °C for 7 min, 35 cycles of 94 °C for 60 s, 53 °C for 120 s, and 72 °C for 180 s. Following the last cycle, there was a 7 min incubation at 72 °C (19). Subsequently, the PCR products were resolved on 1% (*w*/*v*) agarose gels in 1 × Tris-acetate-EDTA (TAE) buffer. The bands in the agarose gels were visualized using the SafeView™ Classic stain (ABM, Richmond, BC, Canada) in the Infinity Gel Imaging System (Vilber Lourmat, Marne-la-Vallée, France).

#### 2.3.3 Detection of some non-typhoidal *Salmonella* serotypes by mPCR

The mPCR method was used to detect some of the non-typhoidal *Salmonella* types. It was aimed to identify *S.* Enteritidis-specific *SdfI*; *S. typhimurium*-specific *fliC-i*; *S.* Dublin-specific *fliC-gp*, and *S.* Stanleyville-specific *fliC-z4,z23* gene regions (Table 1).

**Table 1:** Primers for detection of non-typhoidal *Salmonella* types by mPCR.

Name of primer	Primer Sequence (5′ to 3′)	Size (bp)	Reference
SdfF	TGTGTTTTATCTGATGCAAGAGG	333	(63)
sdfR	CGTTCTTCTGGTACTTACGATGAC		
H-for	ACTCAGGCTTCCCGTAACGC		(20)
Hgp	ATTAACATCCGCCGCGCCAA	779	(20)
Hi	ATAGCCATTITACCAGTICC	551	(64)
H2z4,223F	TTTGTCAAAGATGTTACTGCG	427	(20)
H2z4,223R	AGGTTAGTGATGGCAGATTC		

The mPCR master mix was made according to Tennant et al. (20). The cycling parameters of the mPCR involved denaturation at 95 °C for 2 min, followed by 25 cycles comprised of heating to 95 °C for 30 s, 64 °C for 30 s, and 72 °C for 15 s, and a final step of 72 °C for 5 min.

#### 2.3.4 Agglutination test for investigation of *Salmonella* serovars

Serovars of *Salmonella* isolates were confirmed using the slide agglutination test by identifying the types of the O (SSI Diagnostica, Denmark) and H (SSI Diagnostica, Denmark) antigens with diagnostic sera for *Salmonella* according to the Kauffmann-White scheme (21). The test was conducted at the National Food Reference Laboratory of the Ministry of Agriculture and Forestry of the Republic of Türkiye and the National Enteric Pathogens Reference Laboratory of the General Directorate of Public Health of the Ministry of Health of the Republic of Türkiye.

#### 2.3.5 Sanger sequencing

Following genomic DNA extraction, amplification was performed using the forward 5’-AGAGTTTGATCCTGGCTCAG-3′ and reverse 5’-CTACGGCTACCTTGTTACGA-3′ primers (22) specific to the 16S rRNA region, which were used to amplify an approximately 1,500-base gene region by PCR. Readings obtained from Sanger sequencing primers were assembled into a contig to create a consensus sequence.

The CAP contig assembly algorithm in BioEdit software was used for this process. Sequence analysis results were evaluated using BLAST analysis and similarity scores obtained from NCBI GenBank. Sequence analysis results were evaluated using BLAST analysis and similarity scores obtained from NCBI GenBank. These findings gave clues about the phylogenetic relationships of the samples studied. Additionally, the high similarity scores indicated a close genetic relationship with known species, highlighting the potential for further research in this area.

#### 2.3.6 Phylogenetic tree construction and bioinformatics analysis

**Figure 1:**
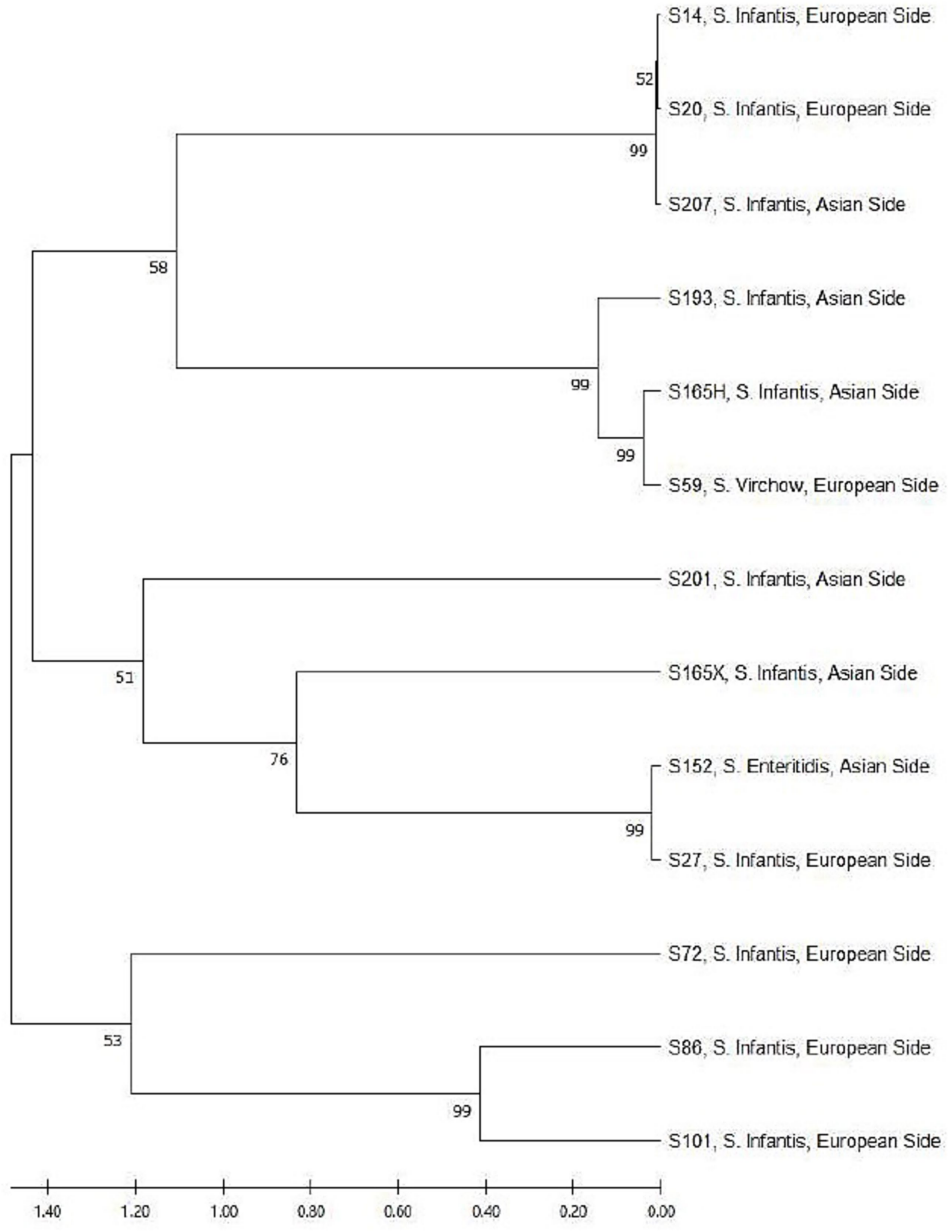
Sanger sequence-based phylogenetic tree of *Salmonella enterica* strains from raw chicken meat.

After sequencing, 16S rRNA sequences were used for homology search and phylogenetic analysis using NCBI-BLAST.^1^ The Phylogenetic Tree for the sequences of the respective strains was constructed using MEGA-11 Software (Figure 1).

### 2.4 Antibiotic susceptibility tests in *Salmonella* serotypes

#### 2.4.1 Screening for antibiotic susceptibility using disc diffusion tests

The antibiotic susceptibility of the isolated *Salmonella* serotypes was tested using agar disc diffusion on Mueller–Hinton Agar (MHA; Oxoid CM 337), in accordance with the European Committee on Antimicrobial Susceptibility Testing (23). Results were interpreted following EUCAST (24) and CLSI (25) guidelines. The disc diffusion test was performed on MHA for 13 different antibiotics (Oxoid, Basingstoke, United Kingdom): kanamycin (CT0026B, 30 μg), gentamicin (CT0024B, 10 μg), cefotaxime (CT0166B, 30 μg), cefazolin (CT0011B, 30 μg), azithromycin (CT0906B, 15 μg), ciprofloxacin (CT0425B, 5 μg), pefloxacin (CT0661B, 5 μg), ampicillin (CT0003B, 10 μg), amoxicillin-clavulanic acid (CT0223B, 30 μg), chloramphenicol (CT0013B, 30 μg), trimethoprim/sulfamethoxazole (CT0052B, 1.25/25 μg), tetracycline (CT0054B, 30 μg), and imipenem (CT0455B, 10 μg). *Escherichia coli* ATCC 25922 served as the quality control strain. Petri dishes were examined after 18 ± 2 h of incubation at 37 °C, and *Salmonella* serotypes were categorized as sensitive or resistant based on zone diameter breakpoints for each antimicrobial, according to EUCAST (24) and CLSI (25) guidelines. For tetracyclines and azithromycin, only CLSI (25) provided breakpoints, while EUCAST (24) did not.

#### 2.4.2 Detection of minimum inhibitory concentration in resistant *Salmonella* serotypes

E-test strips were used to measure the minimum inhibitory concentration of *Salmonella* serotypes according to the EUCAST (24) and CLSI (25) breakpoint tables; only the antibiotic resistance was determined by a disk diffusion test. Accordingly, test strips were used to cefazolin (Liofilchem, 92,174), pefloxacin (Liofilchem, 92,041), azithromycin (Liofilchem, 92,030), ciprofloxacin (Liofilchem, 920,451), trimethoprim/sulphamethoxazole (Liofilchem, 92,123), kanamycin (Liofilchem, 92,034), chloramphenicol (Liofilchem, 92,075), tetracycline (Liofilchem, 92,114), cefotaxime (Liofilchem, 92,007), and amoxicillin-clavulanic acid (Liofilchem, 92,024).

#### 2.4.3 Double disk synergy test for phenotypic confirmation of ESBL production

Double disc synergy test (DDST) was used for ESBL phenotypic confirmation test. CAZ Oxoid, CTO412B, 30 μg, CTX, and AMC antibiotic disks were plated 20 mm apart on MHA with the AMC disc in the middle. The plates were incubated at 37 °C for 24 h (26).

#### 2.4.4 Investigation of ESBLs genes by PCR

The PCR assay was conducted to determine whether the isolates have *bla*_SHV,_ *bla*_TEM,_ *bla*_CTX-M,_ and *bla*_OXA._ PCR mix was as follows (final 25 μL): 2.5 μL DNA samples, 10X KCL buffer 2.5 μL, dNTP mix 2.5 μL, MgCl_2_ 1.5 μL, each primer 0.5 μL, Taq DNA polymerase (Thermo Fisher EP0404, United States) 0,4 μL and dH_2_O 12 μL. mPCR to detect ESBL’s genes condition follows initial denaturation at 95 °C for 15 min, followed by 30 cycles of 94 °C for 30 s, 62 °C for 90 s, and 72 °C for 60 s, and with a final extension at 72 °C for 10 min in the thermal cycle (Applied Biosystems, Veriti, USA) (27). The amplified PCR products were subjected to electrophoresis at a 1.5% agarose gel with a 5 μL safe view (Abm, Richmond, Canada) (Table 2). *Klebsiella pneumoniae* NCTC 13443 metallo beta-lactamase NDM-1 was used as a reference strain.

**Table 2:** Primers and target genes detection of ESBLs and carbapenem-resistance genes by mPCR.

Amplicon	Primer sequence (5′—›3′)	Size (Bp)	Reference
*bla*_SHV_	F-CTTTATCGGCCCTCACTCAA-	237	(27)
	R -AGGTGCTCATCATGGGAAAG-		
*bla*_TEM_	F-CGCCGCATACACTATTCTCAGAATGA	445	(29)
	R-ACGCTCACCGGCTCCAGATTTAT		
*bla*_CTX-M_	F-ATGTGCAGYACCAGTAARGTKATGGC	593	(65)
	R-TGGGTRAARTARGTSACCAGAAYCAGCGG		
*bla*_OXA_	F-ACACAATACATATCAACTTCGC	813	(66)
	R-AGTGTGTTTAGAATGGTGATC		
*bla*_OXA-48_	F- TTGGTGGCATCGATTATCGG	744	(67)
	R- GAGCACTTCTTTTGTGATGGC		
*bla*_NDM_	F-TGGCAGCACACTTCCTATC	488	(67)
	R- AGATTGCCGAGCGACTTG		
*bla*_KPC_	F- CTGTCTTGTCTCTCATGGCC	796	(67)
	R- CCTCGCTGTRCTTGTCATCC		
*bla*_VIM_	F- AGTGGTGAGTATCCGACAG	212	(67)
	R- TCAATCTCCGCGAGAAG		
*bla*_CTX-M_ Group1	F-GCGTGATACCACTTCACCTC	260	(28)
	R-TGAAGTAAGTGACCAGAATC		
*bla*_CTX-M_ Group2	F-TGATACCACCACGCCGCTC	341	(28)
	R-TATTGCATCAGAAACCGTGGG		
*bla*_CTX-M_ Group 8 and 25/26	F-CAATCTGACGTTGGGCAATG	207	(28)
	R-ATAACCGTCGGTGACAATT		
*bla*_CTX-M_ Group9	F-ATCAAGCCTGCCGATCTGGTTA	293	(28)
	R-GTAAGCTGACGCAACGTCTGC		

The PCR assay was conducted to determine whether the isolates have *bla*_CTX-M_ subgroup (1–2-8, 25/26, 9) (28). The amplified PCR products were subjected to electrophoresis at a 1.5% agarose gel with a 5 μL safe view (Abm, Richmond, Canada) (Table 2).

### 2.5 Investigation of carbapenem resistance genes by PCR

Carbapenem resistance genes (*bla*_OXA-48_, *bla*_VIM_, *bla*_NDM_, *bla*_KPC_) were investigated using monoplex PCR. The PCR mix was as follows (final 25 μL): 3 μL DNA samples, 10X KCL buffer 2.5 μL, dNTP mix (dATP, dCTP, dGTP, and dTTP) 2.5 μL, MgCl_2_ 1.5 μL, each primer 0.5 μL, Taq DNA polymerase (Thermo Scientific, United States) 0.14 μL, and dH_2_O 12 μL. *Klebsiella pneumoniae* NCTC 13443 metallo beta-lactamase NDM-1 was used as a reference strain.

The PCR assay conditions were as follows: initial denaturation at 94 °C for 5 min, followed by 30 cycles of 94 °C for 30 s, melting temperature found for 30 s, and 72 °C for 60 s, with a final extension at 72 °C for 10 min in the thermal cycler (Applied Biosystems, Veriti, USA). Amplificons were subjected to electrophoresis at a 1.5% agarose gel (*w*/*v*) containing 5 μL SafeView (Abm, Richmond, Canada) (29) (Table 2).

### 2.6 Investigation of mobilized colistin resistance genes by mPCR

The PCR assay investigated whether the *Salmonella* serotypes (*n* = 13) contained mobilized colistin resistance genes (Table 3). Conditions of multiplex PCR to detect *mcr*-1, *mcr*-2, *mcr*-3, *mcr*-4, and *mcr*-5 genes were as follows: initial denaturation at 95 °C for 15 min, followed by 30 cycles of 94 °C for 30 s, 58 °C for 90 s, and 72 °C for 60 s; and a final extension at 72 °C for 10 min in the thermal cycler (29). *E. coli* NCTC 13846 was used as the reference strain. Amplification products were analyzed in 1.5% (*w*/*v*) agarose gel containing 5 μL SafeView.

**Table 3:** Primers to detect different mobilized colistin resistance genes (1–5) by mPCR.

Target gene	Primer sequence (5′—›3′)	Melting temperature Tm (°C)	Product size (bp)
*mcr-1*	F- AGTCCGTTTGTTCTTGTGGC	58	320
	R- AGATCCTTGGTCTCGGCTTG		
*mcr-*2	F- CAAGTGTGTTGGTCGCAGTT	58	715
	R- TCTAGCCCGACAAGCATACC		
*mcr*-3	F- AAATAAAAATTGTTCCGCTTATG	58	929
	R- AATGGAGATCCCCGTTTTT		
*mcr*-4	F- TCACTTTCATCACTGCGTTG	58	1,116
	R- TTGGTCCATGACTACCAATG		
*mcr*-5	F- ATGCGGTTGTCTGCATTTATC	58	1,644
	R- TCATTGTGGTTGTCCTTTTCTG		

### 2.7 Statistical analysis

Data on the prevalence of *Salmonella* in chicken parts were analyzed using Pearson’s chi-square and Fisher’s exact tests for independence to determine whether *Salmonella* prevalence differed across types and regions in Istanbul, Türkiye (*α* = 0.05).

## 3 Results

### 3.1 Detection of *Salmonella* in chicken meat samples in Istanbul and phylogenetic relationship

In this study, 14 raw chicken meat samples were found to be positive for *Salmonella* isolates analyzed using the conventional ISO method (17). In total, 92.9% (13/14) of *Salmonella* isolates confirmed by PCR. 1 isolate was identified as *S.* Enteritidis by mPCR, but the serotype of the other *Salmonella* isolates could not be determined. Therefore, the agglutination test and Sanger sequencing methods were preferred for molecular confirmation. These *Salmonella* 13 isolates were serotyped by Sanger sequencing, and *S.* Infantis 11/13 (84.6%) was the dominant serotype. In addition, one (7.7%) *Salmonella* isolate serotyped as *S.* Virchow, and the other (7.7%) *Salmonella* strains were *S.* Enteritidis (Table 4).

**Table 4:** Distribution of *Salmonella* serotypes in chicken parts from the Asian and European sides of Istanbul.

Side of Istanbul	Drumstick	Wing	Thigh	Breast
Europe	6.6% (1/15) *S.* Infantis	12.5% (5/40) *S.* Infantis 2.5% (1/40) *S.* Virchow	0	0
Asia	0	13.8% (5/29) *S.* Infantis	3.7% (1/27) *S.* Enteritidis	0

The *S.* Infantis strain obtained from the Asian side and the *S.* Virchow strain obtained from the European side were phylogenetically very close (Figure 1).

### 3.2 Antibiotic susceptibility tests in *Salmonella* serotypes

In this study, a disc diffusion test was performed to assess antibiotic susceptibility in *Salmonella* strains. 13 *Salmonella* serotypes were resistant to cefazolin, and 92.3 and 84.6% were resistant to pefloxacin (a fluoroquinolone) and tetracycline, respectively, according to the CLSI (25). However, all strains were susceptible to ampicillin, imipenem, and gentamicin by EUCAST (24) and CLSI (25) guidelines (Table 5).

**Table 5:** Clinical and laboratory standards institute (CLSI) and the European committee on antimicrobial susceptibility testing (EUCAST) as assessed by the disc diffusion method of *Salmonella* serotypes (*n* = 13) [Resistant (“R”); or susceptible (“S”)].

Antibiotic groups	Name of antibiotics	Distribution of *Salmonella* isolates according to CLSI (25)		Distribution of *Salmonella* isolates according to EUCAST (24)	
		R (%)	S (%)	R (%)	S (%)
Aminoglycoside	Kanamycin 30 μg	30.8% (*n* = 4)	69.2% (*n* = 9)	*–	*–
	Gentamicin 10 μg	0	100% (*n* = 13)	0	100% (*n* = 13)
Cephalosporins	Cefotaxime 30 μg	15.4% (*n* = 2)	84.6% (*n* = 11)	0	100% (*n* = 13)
	Cefazolin 30 μg	100% (*n* = 13)	0	100% (*n* = 13)	0
Macrolid	Azithromycin 15 μg	76.9% (*n* = 10)	23.1% (*n* = 3)	*–	*–
Fluoroquinolones	Ciprofloxacin 5 μg	7.6% (*n* = 1)	92.4% (*n* = 12)	30.7% (*n* = 4)	69.3% (*n* = 9)
	Pefloxacin 5 μg	92.4% (*n* = 12)	7.6% (*n* = 1)	84.6% (*n* = 11)	15.3% (*n* = 2)
Penicillin	Ampicillin 10 μg	0	100% (*n* = 13)	0	100% (*n* = 13)
	Amoxicillin clavulanic acid 30 μg	0	100% (*n* = 13)	7.6% (*n* = 1)	92.4% (*n* = 12)
Phenicol	Chloramphenicol 30 μg	7.6% (*n* = 1)	92.4% (*n* = 12)	7.6% (*n* = 1)	92.4% (*n* = 12)
Sulfonamides	Trimethoprim-Sulfamethoxazole 25 μg	15.4% (*n* = 2)	84.6% (*n =* 11)	15.3% (*n* = 2)	84.6% (*n* = 11)
Tetracyclines	Tetracycline 30 μg	84.6% (*n* = 11)	15.3% (*n* = 2)	*–	*–
Carbapenem	Imipenem 10 μg	0	100% (*n* = 13)	0	100% (*n* = 13)

*–: The EUCAST (2022) breakpoint table testing does not provide a breakpoint value for this antibiotic.

### 3.3 Detection of MIC in resistant *Salmonella* serotypes

*Salmonella* strains demonstrating antibiotic resistance in the disc diffusion assay were selected for E-Test testing to determine MICs. Based on MIC results, the highest tetracycline resistance among *Salmonella* strains was 84.6%. Resistance to tetracycline is followed by resistance to macrolides (53.8%) and aminoglycosides (38.4%). Interestingly, the disc diffusion test showed that all strains were resistant to cefazolin; however, the MIC test did not support this (Table 6).

**Table 6:** The MIC, as assessed by E-test, for 10 antimicrobial agents against *Salmonella* serotypes.

Group	Name of antibiotics	MIC (μg/mL), *n* = 13										Resistant isolates	
		0.012–0.025	0.026–0.50	0.051–0.999	1–2	8	12–16	32	48	64	125	>256	
Cephalosporins	Cefazolin	–	–	8	4	–	–	–	–	–	–	–	0
	Cefotaxime	2	–	–	–	–	–	–	–	–	–	–	0
Aminoglycosides	Kanamycin	–	–	–	–	–	–	–	–	–	–	5	**5**
Fluoroquinolones	Ciprofloxacin	4	–	–	–	–	–	–	–	–	–	–	**2**
	Pefloxacin	–	–	–	4	3	3	–	–	–	–	1	–
Penicillin	Amoxicillin clavulanic acid	–	1	–	–	–	–	–	–	–	–	–	0
Macrolid	Azithromycin	–	–	–	–	–	–	–	–	–	–	5	**7**
Phenicol	Chloramphenicol	–	–	–	–	–	–	–	–	–	–	1	**1**
Sulfonamides	Trimethoprim-Sulfamethoxazole	–	1	1	–	–	–	–	–	–	–	–	0
Tetracyclines	Tetracycline	–	–	–	–	–	–	–	–	–	–	10	11

The present study detected MDR in 72.7% of *S.* Infantis strains. However, MDR was not determined in *S.* Enteritidis and *S.* Virchow strains. MIC values were prioritized in the MDR detected. 75% (6/8), 12.5% (1/8), and 12.5% (1/8) MDR *S.* Infantis strains were isolated from the wing, drumstick, and thigh, respectively. Additionally, MDR *S.* Infantis strains were equally distributed in the European and Asian sides of Istanbul (Table 7).

**Table 7:** Antibiotic resistance profiles of *Salmonella* strains.

Sample no	Part of Istanbul	Serovar	ESBL gene contained in the strain	Antibiotic resistance patterns
S14	Europe	*S.* Infantis	–	*AZM, **TE, ***PEF
S20	Europe	*S.* Infantis	*bla*_TEM_	**TE, ****K, ***PEF
S27	Europe	*S.* Infantis	–	*AZM, **TE, ****K, ***PEF
S59	Europe	*S*. Virchow	*bla*_TEM_	-
S72	Europe	*S.* Infantis	–	**TE, ***PEF,
S86	Europe	*S.* Infantis	*bla*_CTX-M_	**TE
S101	Europe	*S.* Infantis	–	*AZM, **TE, ***PEF
S152	Asia	*S.* Enteritidis	*bla*_TEM_, *bla*_CTX-M_	-
S165X	Asia	*S.* Infantis	–	*AZM, **TE, ****K
S165H	Asia	*S.* Infantis	*bla*_CTX-M_	**TE, ****K, ***PEF
S193	Asia	*S.* Infantis	*bla*_TEM_, *bla*_CTX-M_	AZM, **TE, *****K, *****C, ***PEF
S201	Asia	*S.* Infantis	–	**TE, ****K, ***PEF
S207	Asia	*S.* Infantis	–	*AZM, **TE, ***PEF

*AZM: Azithromycin; **TE: Tetracycline; ***PEF: Pefloxacin; ****K: Kanamycin; *****C: Chloramphenicol.

### 3.4 ESBL-producing *Salmonella*, carbapenem, and colistin resistance in *Salmonella* serotypes

As a result of DDST used for phenotypic confirmation of ESBL production, 38% of isolates were found to produce ESBLs. As a result of this study, 30.7% of *Salmonella* serotypes containing the *bla*_TEM_ gene by PCR, *bla*_OXA-2_, and *bla*_SHV_ were not detected. These strains were *S.* Infantis (2), *S.* Virchow, and *S.* Enteritidis. As a result of the examination of resistance genes related to the *bla*_CTX-M_ subgroups (1, 2, 8, 9, and 25/26) by mPCR, the *bla*_CTX-M_ gene of subgroup 1 was detected in 1 strain (7.7%), while the gene of subgroup 9 was identified in 4 strains (30%). PCR investigated carbapenem and colistin resistance in *Salmonella* serotypes, and the presence of *bla*_OXA-48_, *bla*_VIM_, *bla*_NDM_, *bla*_KPC_, *mcr*-1, *mcr*-2, *mcr*-3, *mcr*-4, and *mcr*-5 genes was analyzed. Carbapenem resistance using the disc diffusion method with imipenem also investigated. In our study, no *Salmonella* serotypes were included in the relevant genes, carbapenem and colistin resistance were not detected in any strains. Additionally, imipenem-resistant *Salmonella* strains were also not determined.

## 4 Discussion

The worldwide consumption of chicken meat is relatively high due to economic, religious reasons, and the popularity of chicken products. As a result, chicken meat is favored in many food businesses and restaurants (30). A total of 6.2% (13/210) of *Salmonella* strains were isolated from chicken meat samples: the wing (76.9%; 10/13), thigh (15.3%; 2/13), and drumstick (7.7%; 1/13). *Salmonella* was not found in any breast samples in this study. Similarly, Sezen et al. (31) reported 6% of *Salmonella* spp. in chicken meat samples from Istanbul. In another study, 3% of *Salmonella* was detected in chicken meat samples, with 1% found in wings and drumsticks in Türkiye (32). A study in Brazil found 46.1% (53/115) of *Salmonella* spp. on chilled chicken meat collected at retail points (33). Perin et al. (34) also reported 31.5% *Salmonella* in chicken meat samples from Brazil. Conversely, Pavelquesi et al. (33) and Perin et al. (34) observed higher rates of *Salmonella* in chicken wings compared to other parts. Given that both Brazil and Türkiye are large chicken meat producers, understanding *Salmonella* epidemiology in these countries is important. The differences in *Salmonella* prevalence reported across studies may also result from variations in detection methods (35).

A previous study reported *Salmonella* spp. in 15% of 100 raw chicken meat samples collected from Istanbul, with *S.* Enteritidis making up a relatively high proportion (26.6%) of the isolates (30). The findings of our study, along with those of reference (30), provide important insights into *Salmonella* isolation and identification in similar samples from the same province. Additionally, the number of *Salmonella* serotypes originating from chicken meat was higher on the European side of Istanbul (53.9%; 7/13) compared to the Asian side (46.1%; 6/13). Similarly, another study found that *Salmonella* prevalence in raw chicken carcasses was higher on the European side of Istanbul (53.3%; 8/15) (30). This difference may be due to the increasing population density on the European side and inadequate hygiene practices at points of sale.

Rapid molecular methods for detecting *Salmonella* include PCR-based assays and next-generation sequencing (NGS) (36). In a study, 21.2% of *Salmonella* strains were detected in raw chicken meat (24/113) collected from markets in Korea. Through of molecular analysis (MLST), they identified *S.* Enteritidis as the dominant strain (45.8%), followed by *S.* Virchow (25%), *S.* Montevideo (8.3%), *S.* Bsilla (8.3%), *S.* Bareilly (4.2%), *S.* Dessau (4.2%), and *S.* Albany (4.2%) (9). Previously, many researchers also reported *S.* Enteritidis as the most common species in chicken products (30, 35, 37, 38). However, in the present study, *S.* Infantis was identified as the dominant species. Similarly, recent studies have noted an increased prevalence of serotypes such as *S.* Infantis (3, 15, 39). Since 2011, *S.* Infantis has been reported as the fourth leading cause of Salmonellosis in humans (40). Additionally, *S.* Infantis was among the most frequently identified *Salmonella* species in the EU, accounting for 36.5% in chicken and 36.5% in other animal meat (41). These European findings are consistent with our study results. Moreover, it is noteworthy that *Salmonella* spp. (6.2%) was close to European data (7.3%) (8) and species-level data in our study. Although *S.* Infantis does not typically infect poultry, it remains a public health concern because this serotype can cause disease in humans (42).

The *S.* Infantis strain sourced from the Asian region and the *S*. Virchow strain sourced from the European region exhibited significant phylogenetic proximity. Starting in summer 2017, the *S.* Virchow outbreak continued in EU countries, and *S.* Virchow was monitored as a public health threat to poultry farms. As a result of the WGS analysis performed on this strain, it was reported that chicken meat-origin strains were predominant. Furthermore, comparing representative outbreak strains with existing *S.* Virchow ST16 genome profiles from non-human isolates showed that most matching isolates originated from broilers in Germany, the Netherlands, and France. Most human cases have been reported to have originated at local kebab restaurants. The hospitalization rate for cases caused by this agent was 38.5% in Germany, underscoring the importance of monitoring chicken meat throughout the food chain (43). Similarly, there was a very close genetic affinity between the ESBL-producing *S.* Enteritidis strain isolated from the Asian side and the *S.* Infantis strain isolated from the European side. The other ESBL-producing strain, *S.* Infantis (S20), was phylogenetically more distant from other ESBL-producing strains.

Antibiotic resistance is a significant public health concern because it can spread through food, from animals to humans, or via cross-contamination between different sources (11). *Salmonella* is one of the most important antibiotic-resistant zoonotic pathogens and serves as an indicator bacterium of food, human, and animal origin (13). In this study, the disc diffusion method was used to determine the antibiotic susceptibility of *Salmonella* strains. All isolates were resistant to cefazolin, while 92.3 and 84.6% showed resistance to pefloxacin (a fluoroquinolone) and tetracycline, respectively, according to CLSI guidelines (25). By contrast, all strains were susceptible to ampicillin, imipenem, and gentamicin, based on EUCAST (24) and CLSI (25) standards.

Comparable findings have been reported elsewhere. For instance, high resistance rates to trimethoprim–sulfamethoxazole were found in *Salmonella* strains isolated from chicken meat, with 61.2% in Iran (35) and 71.4% in Türkiye (44). Another study documented complete resistance to amoxicillin (100%). In comparison, resistance to cefotaxime (55.1%) and chloramphenicol (42.4%) was moderate, and no resistance to kanamycin (0%) was observed, contrasting with the resistance profile seen in our study (16). Pavelquesi et al. (33) found resistance to amoxicillin/clavulanic acid (83.3%), sulfonamides (64.1%), tetracycline (46.2%), and ciprofloxacin (65.4%) in *Salmonella* isolates from chicken meat in Brazil, using disc diffusion tests. Our study reported high tetracycline resistance (84.6%) in *Salmonella* strains, with comparable rates of fluoroquinolone resistance similar to Wang et al. (45). Conversely, tetracycline, amoxicillin-clavulanic acid, and ciprofloxacin resistance in *E. coli* (*Enterobacterales*) from chicken meat in Istanbul were 74.2, 87.1, and 45.5%, respectively (46). It is important to note that both studies were conducted in Istanbul within the same year, which is essential when monitoring antibiotic-resistant bacteria in chicken meat.

Rincón-Gamboa et al. (47) reported that tetracycline resistance was the most frequently observed through MIC tests. Additionally, researchers noted high resistance to cephalosporins and ampicillin, as determined by MIC. It is beneficial for public health that cephalosporin resistance, which is closely associated with extended-spectrum beta-lactamase, was not detected in the current study. Conversely, Rincón-Gamboa et al. (47) identified different serotypes of *S.* Infantis in most of the studies they examined, which aligns with our findings. Furthermore, Rau et al. (48) analyzed *Salmonella* in poultry meat in Brazil during 2014 and 2017 and found evidence of antibiotic resistance, particularly in *Salmonella* ser. Heidelberg and *Salmonella* ser. Minnesota. Resistance rates in 2014 and 2017 to cefotaxime (76/146, 52.1% and 124/163, 76.1%), ciprofloxacin (83/146, 56.9% and 145/163, 89.0%), and tetracycline (88/146, 60.3% and 135/163, 82.8%) were also identified by MIC testing. While tetracycline resistance was similar across studies, there was a significant difference in cephalosporin resistance rates compared to our results.

Multi-drug-resistant *Enterobacterales* constitute a significant health problem that threatens global health and is becoming increasingly difficult to treat. MDR-containing foodborne pathogens are instrumental in spreading antibiotic resistance and resistance from farm animals to the general public (11). The present study detected MDR in 72.7% of *S.* Infantis strains. Lee et al. (49) isolated *S.* Kentucky (25.58%), *S.* Reading (18.60%), *S.* Infantis (11.63%), and *S. typhimurium* (9.30%) in their study on 958 retail meat samples in the United States. 13.95% of the strains (*n* = 6) were found to contain MDR, and the distribution of these strains were *S.* Infantis (*n* = 4), *S.* Reading (*n* = 1), and *S.* Kentucky (*n* = 1). Similar to our results, tetracycline (52.17%) was the most resistant group in the MDR *Salmonella* strains isolated from chicken meat. Conversely, Lee et al. (49) reported that all strains were susceptible to azithromycin. Moreover, they noted that one *S.* Kentucky strain was resistant to amoxicillin-clavulanic acid, cefoxitin, and ceftriaxone. The fact that cephalosporin resistance, which is associated with ESBL production and is a significant issue in MDR, was not found in the present study is valuable for public health.

Chen et al. (50) found that *S. Corvallis*, *S.* Kentucky, and *S.* Agona were the dominant species among *Salmonella* isolates from pork, duck, and chicken meat offered for retail sale in South China. 80.1% of the *Salmonella* strains contained MDR. In addition, they stated that the MDR rate was incredibly high (91.8%) in *S.* Kentucky strains. Moreover, tetracycline (93.8%) was reported as the prominent antimicrobial agent. On the other hand, Wang et al. (51) reported that *S. typhimurium* (23.73%, 8,397/35,382) is the most prevalent serovar in both human and non-human sources in China, followed by *S.* Enteritidis, *S.* Derby, *S.* London, and *S.* Thompson. Researchers have identified ESBL-resistance, mobile colistin resistance, fosfomycin resistance, and mobile tigecycline resistance genes in MDR *Salmonella* strains (>45%). Another study found an MDR rate of 53.8% among *Salmonella* strains isolated from chilled chicken meat in Brazil (33). The most common antibiotic resistance rates were determined against amoxicillin/clavulanic acid (83.3%), followed by sulfonamide (64.1%) and tetracycline (46.2%) in MDR *Salmonella* strains. It has been observed that different serotypes are common in *Salmonella* strains from poultry meat reported from other continents, such as Asia (52, 53) and Brazil (34), and Europe (43), and that MDR to different antibiotics is higher in parallel. Researchers reported mainly tetracycline and sulfanamide resistance in *Salmonella* serotypes (33, 35, 43, 47). On the other hand, it is essential to note that cephalosporin resistance detected in ESBL-producing *Salmonella* strains was not observed in the present study, unlike other studies. Indeed, Aydin et al. (46) detected 79.2% MDR *E. coli* in raw chicken meat obtained from Istanbul in the same year as this study. They reported high resistance rates to cephalosporins, penicillins, and sulfonamides, although similar tetracycline resistance was also detected. Therefore, the characteristics of MDR *Salmonella* need to be investigated in more comprehensive studies.

As a result of DDST used for phenotypic confirmation of ESBL production, 38% of isolates were found to produce ESBLs. Kahraman et al. (54) reported a 37.5% rate of phenotypic ESBL production in *Salmonella* strains isolated from chicken carcasses collected in Istanbul. Unlike the present study, they did not detect any *bla*_CTX-M_ genes. Notably, there is an approximate 7-year gap between the two studies’ sample collection periods. These findings suggest a rising trend in antimicrobial resistance over time, likely driven by continued antibiotic use. Additionally, the potential for antibiotic resistance conferred by mobilized genetic elements, such as plasmids, is a concern. This suggests that, while resistance rates may currently be undetectable, they could increase in the future. In this context, Aydin et al. (46) reported a high rate of ampicillin resistance (78.2%) and the presence of the *bla*_TEM_ gene associated with this resistance (97.02%). Suleymanoglu et al. (55) reported a similar result for the *bla*_TEM_ gene in all *E. coli* isolates, detecting 80% ampicillin resistance. The mobility of these genes on transposons and plasmids could further accelerate their spread and thus pose a potential threat to human health (44). Li et al. (56) reported that ampicillin resistance was 97.5% and that *bla*_TEM_ was detected in 100% of 40 *Salmonella* strains isolated from 200 chicken carcasses in China. They detected a very high rate of ESBL-producing *Salmonella*. Kang et al. (57) isolated a 1.4% strain from 555 chicken meat samples in South Korea. These strains were identified as *S.* Enteritidis and differed from this study by carrying the *bla*_CTX-M-1_ gene and ampicillin resistance. Although no ampicillin resistance in *Salmonella* serotypes was observed in this study, the detection of the blaTEM gene at a 30% rate indicates that plasmid-mediated resistance has a complex epidemiology.

Wang et al. (51) reported that 59.9% of ESBL-producing *Salmonella* strains were CTX-M type beta-lactamases. Another study, Adigüzel et al. (58), conducted in 2016, found that 29.9% of *Salmonella* strains from retail chicken meat in Türkiye produced ESBL. They investigated the same *bla*_CTX-M_ subgroups as this study and found that the *bla*_CTX-M-8-25_ gene was dominant. Although both studies were conducted in the same country, they took place in different regions, which may have contributed to the differences in *bla*_CTX-M_ subgroup. Additionally, there is about a 5 year gap between the sample collection years of the two studies, and it appears that different *bla*_CTX-M_ subgroups have become more prominent durings period.

Carbapenem and colistin are essential treatment options as antibiotics of last resort. It has been observed that Enterobacterales develop resistance to these antibiotics more frequently. ESBL-producing and carbapenem-resistant Enterobacterales can increase colistin use over time despite its nephrotoxic and neurotoxic effects and its narrow spectrum of activity (11). WHO (12) reports carbapenem-resistant and colistin-resistant Enterobacterales as a critical global public health threat. Conversely, Jeon et al. (59) investigated imipenem resistance in *Salmonella* strains isolated from retail chicken meat using disc diffusion and reported that 1.8% of the strains were resistant. In another study, imipenem and carbapenem resistance were examined, and 0.2% of carbapenem-resistant *Salmonella* strains were isolated from individuals under 18 (60). Li et al. (61) examined the presence of the mcr-1 gene in *Salmonella* strains from retail food products and food poisoning cases. While they did not find mcr-1 in 950 *Salmonella* strains collected between 2006 and 2011, they reported that 3% of 333 *Salmonella* strains isolated in 2012–2015 contained *mcr*-1. Casagrande Proietti et al. (62) analyzed colistin resistance in 85 *Salmonella* Infantis strains, and 3.5% showed phenotypic colistin resistance (MIC test). The mcr-1.1 variant was found in one *Salmonella* Infantis strain, and the *mcr*-1.2 variant in two strains, using WGS and PFGE methods. The fact that carbapenem- and polymyxin-group antibiotics are not frequently used in the poultry sector in Türkiye may explain the absence of resistance to these groups.

Foodborne salmonellosis cases pose a threat to public health, and inadequate heat treatment along with cross-contamination in poultry meat and meat products increase this risk. This study identified *Salmonella* serotypes from chicken meat that can be particularly harmful to human health. For risk assessment, the likelihood of *Salmonella*-related foodborne illness was considered low because the overall consumption frequency of chicken was very low, regardless of consumption patterns or cooking methods. The study noted that *S.* Infantis was the dominant serotype isolated from chicken meat in Istanbul. This finding aligns with recent studies conducted in Türkiye and neighboring countries in the Balkan region, Europe. However, a shift in the dominant *Salmonella* serotype could pose new health risks compared to previous research. Despite growing concerns about foodborne illnesses caused by *Salmonella* from poultry consumption, many studies in Türkiye suggest that the risk remains relatively acceptable. Additionally, *Salmonella* serotypes showed resistance to several antibiotics used to treat salmonellosis. They also complicate treatment by spreading antibiotic resistance through mobile genetic elements such as plasmids. The detection of ESBL-producing *Salmonella* strains presents a serious public health threat. The rates of carbapenem and colistin-resistant *Salmonella* strains are lower than those observed in *E. coli* and *K. pneumoniae*, other members of Enterobacterales, which show higher resistance rates. It is crucial for public health to avoid the unnecessary use of last-resort antibiotics like carbapenems and colistin and to monitor resistance development. Finally, the study found that the rate of MDR *S.* Infantis was relatively high. Therefore, adopting the ‘One Health’ approach is essential to prevent further public health damage by controlling antibiotic use.

## Data Availability

The datasets presented in this study can be found in online repositories. The names of the repository/repositories and accession number(s) can be found at: https://www.ncbi.nlm.nih.gov/, CP193634.1; https://www.ncbi.nlm.nih.gov/genbank/, CP193634.1.

## Footnotes

1
https://blast.ncbi.nlm.nih.gov/Blast.cgi

## Author’s note

This study is based on the Ph.D. thesis of the first author, Selman Bahadır Orhan.
